# Analysis of serum peptidome profiles of non-metastatic and metastatic feline mammary carcinoma using liquid chromatography-tandem mass spectrometry

**DOI:** 10.1186/s12917-024-04148-y

**Published:** 2024-06-29

**Authors:** Weejarin Paphussaro, Sittiruk Roytrakul, Narumon Phaonakrop, Wannapol Buthasane, Anudep Rungsipipat, Theerawat Tharasanit, Gunnaporn Suriyaphol

**Affiliations:** 1 Biochemistry Unit, Department of Physiology, Faculty of Veterinary Science, Chulalongkorn University, Henri Dunant Road, Pathumwan, Bangkok, 10330 Thailand; 2 Center of Excellence for Companion Animal Cancer, Faculty of Veterinary Science, Chulalongkorn University, Henri Dunant Road, Pathumwan, Bangkok, 10330 Thailand; 3 Functional Proteomics Technology Laboratory, Functional Ingredients and Food Innovation Research Group, National Center for Genetic Engineering and Biotechnology, National Science and Technology Development Agency, 113 Thailand Science Park, Phahonyothin Road, Khlong Nueng, Khlong Luang, Pathum Thani, 12120 Thailand; 4 Department of Pathology, Faculty of Veterinary Science, Chulalongkorn University, Henri Dunant Road, Pathumwan, Bangkok, 10330 Thailand; 5 Department of Obstetrics, Gynaecology and Reproduction, Faculty of Veterinary Science, Chulalongkorn University, Henri Dunant Road, Pathumwan, Bangkok, 10330 Thailand

**Keywords:** Feline mammary gland carcinoma, LC–MS/MS, Metastasis, Serum peptidomics

## Abstract

**Background:**

Feline mammary carcinoma (FMC) is a common aggressive and highly metastatic cancer affecting female cats. Early detection is essential for preventing local and distant metastasis, thereby improving overall survival rates. While acquiring molecular data before surgery offers significant potential benefits, the current protein biomarkers for monitoring disease progression in non-metastatic FMC (NmFMC) and metastatic FMC (mFMC) are limited. The objective of this study was to investigate the serum peptidome profiles of NmFMC and mFMC using liquid chromatography-tandem mass spectrometry. A cross-sectional study was conducted to compare serum peptidome profiles in 13 NmFMC, 23 mFMC and 18 healthy cats. The liquid chromatography-tandem mass spectrometry analysis was performed on non-trypsinized samples.

**Results:**

Out of a total of 8284 expressed proteins observed, several proteins were found to be associated with human breast cancer. In NmFMC, distinctive protein expressions encompassed double-stranded RNA-binding protein Staufen homolog 2 (STAU2), associated with cell proliferation, along with bromodomain adjacent to zinc finger domain 2A (BAZ2A) and gamma-aminobutyric acid type A receptor subunit epsilon (GABRE), identified as potential treatment targets. Paradoxically, positive prognostic markers emerged, such as complement C1q like 3 (C1QL3) and erythrocyte membrane protein band 4.1 (EPB41 or 4.1R). Within the mFMC group, overexpressed proteins associated with poor prognosis were exhibited, including B-cell lymphoma 6 transcription repressor (BCL6), thioredoxin reductase 3 (TXNRD3) and ceruloplasmin (CP). Meanwhile, the presence of POU class 5 homeobox (POU5F1 or OCT4) and laminin subunit alpha 1 (LAMA1), reported as metastatic biomarkers, was noted.

**Conclusion:**

The presence of both pro- and anti-proliferative proteins was observed, potentially indicating a distinctive characteristic of NmFMC. Conversely, proteins associated with poor prognosis and metastasis were noted in the mFMC group.

**Supplementary Information:**

The online version contains supplementary material available at 10.1186/s12917-024-04148-y.

## Background

Feline mammary tumors rank as the third most frequent tumors, following hematopoietic and skin tumors, constituting approximately 17% of all tumors in female cats [1]. Among these, feline mammary carcinoma (FMC) holds the highest prevalence, contributing to 80% of mammary tumors [2]. The conventional tumor, node and metastasis (TNM)-based staging scheme established by the World Health Organization (WHO) has been developed to assess FMC. Concerning metastasis, this disease is frequently associated with ulceration and demonstrates a propensity for regional or distant metastasis, significantly elevating mortality rates, especially in cases involving lung metastasis [3, 4]. Hence, early diagnosis and the implementation of effective treatment play a pivotal role in preventing both local and distant metastasis, contributing significantly to extended survival times [5]. The standard diagnostic procedure for mammary tumors involves biopsy of affected tissues, followed by a comprehensive histopathological examination. This examination is typically carried out subsequent to mastectomy surgery, serving to confirm the presence of cancer [2]. Additionally, various adjuvant chemotherapy protocols have been employed for FMC treatment involving the use of doxorubicin, either alone or in combination with cyclophosphamide [6, 7]. Acquiring molecular data before surgery could mark a crucial turning point in enhancing our understanding of the disease. A number of tissue molecular markers for FMC, including estrogen receptor (ER), progesterone receptor (PR), feline homologue of HER2 (fHER2), cytokeratin 5/6 (CK5/6) and Ki-67, have been reported to classify FMC subtypes [8]. Regarding serum biomarkers, most markers have been focused on identifying HER2-positive FMC [9–11]. In addition, the identification of therapeutic biomarkers holds paramount importance in facilitating effective communication with pet owners. In human breast cancer, bromodomain adjacent to zinc finger domain 2A (BAZ2A) and gamma-aminobutyric acid type A receptor subunit epsilon (GABRE) have been recognized as potential therapeutic targets [12, 13]. The majority of mammary cancer cases in both cats and humans manifest as malignant glandular epithelial tumors, contrasting with the complex and mixed mammary tumors predominantly observed in dogs [14]. Hence, FMC has been proposed as a potential model for studying human breast cancer when compared to its canine counterpart [15, 16]. Efforts have been undertaken to identify and compare prognostic biomarkers in FMC with their human counterparts, specifically in human triple-negative breast cancer, or considering the effects of the tumor microenvironment [15, 16]. However, with the limited available protein biomarkers for monitoring disease progression in non-metastatic FMC (NmFMC) and metastatic FMC (mFMC), it is difficult to categorize FMCs using the same immunophenotypic and molecular biomarkers established for human breast cancer [17, 18].

The serum peptidome consists of low-molecular-weight peptides that can be actively synthesized or proteolytically cleaved from precursor proteins by endogenous proteases [19]. Serum peptidome profiles serves as potential sources for cancer biomarkers. Serum peptidomics has been used to identify late-stage oral melanoma and late-stage oral squamous cell carcinoma in dogs, as well as sarcomeric gene mutation and hypertrophic cardiomyopathy in cat [20, 21]. Moreover, profiles of tumor-suppressive peptide biomarkers for ovarian and breast cancers were identified in humans [22, 23]. Numerous previous studies have employed proteomics approaches to investigate diseases in cat, including the mucosal proteome in cats with inflammatory bowel disease and alimentary small cell lymphoma, as well as the serum proteome in cats with chronic enteropathies [24, 25]. In addition, potential serum biomarkers were discovered using proteomics in canine mammary tumors and canine lymphoma [26–28].

In a previous study, the serum proteome of feline NmFMC was analyzed compared with healthy controls. However, no comparative omics studies of FMC with and without metastasis have been conducted [29]. In this study, serum samples were also utilized to provide molecular information on NmFMC and mFMC, as serum can be easily obtained in routine clinical practice. The aim of this study was to investigate potential peptidome-based serum biomarker profiles for NmFMC and mFMC using liquid chromatography-tandem mass spectrometry (LC–MS/MS).

## Results

### Sample description data

Among the 36 female cats included in the study, 89% were domestic shorthair (32/36), followed by 8% Persian (3/36) and 3% Khao Manee (1/36). The average age of the cats was 10.6 years. Of these, 64% were neutered (23/36), 28% were intact (10/36) and 8% had an unknown status (3/36). Regarding the metastatic status of FMC, 36% were categorized as NmFMC (13/36), while 64% were classified as mFMC (23/36) (Table 1).

**Table 1:** Samples description data: breed, age, neuter status, clinical stage of cancer

Sample ID	Metastasis	Clinical stage	Age (years)	Breed	Neuter status	Metastatic site
1	Nm^a^	I	8	DSH^c^	+	-
2	Nm	I	7	DSH	+	-
3	Nm	II	8	DSH	-	-
4	Nm	II	10	DSH	N/D^d^	-
5	Nm	II	3	Persia	+	-
6	Nm	II	15	DSH	+	-
7	Nm	II	5	DSH	N/D	-
8	Nm	II	8	DSH	N/D	-
9	Nm	III	17	DSH	-	-
10	Nm	III	10	DSH	-	-
11	Nm	III	7	DSH	+	-
12	Nm	III	7	DSH	+	-
13	Nm	III	11	DSH	+	-
14	m^b^	III	12	DSH	+	Lymph node
15	m	III	9	DSH	-	Lymph node
16	m	III	10	DSH	+	Lymph node
17	m	III	13	DSH	+	Lymph node
18	m	lII	11	DSH	-	Lymph node
19	m	III	15	DSH	+	Lymph node
20	m	III	15	DSH	-	Lymph node
21	m	III	8	DSH	+	Lymph node
22	m	III	9	Persia	-	Lymph node
23	m	III	16	DSH	-	Lymph node
24	m	III	14	DSH	+	Lymph node
25	m	III	10	DSH	+	Lymph node
26	m	III	10	DSH	+	Lymph node
27	m	III	15	DSH	+	Lymph node
28	m	III	13	DSH	+	Lymph node
29	m	III	N/D	DSH	+	Lymph node
30	m	III	2	DSH	-	Lymph node
31	m	III	16	Persia	-	Lymph node
32	m	III	17	DSH	+	Lymph node
33	m	IV	5	Khaomanee	+	Lung
34	m	IV	13	DSH	+	Lung
35	m	IV	12	DSH	+	Lung
36	m	IV	10	DSH	+	Lung

^a^*Nm* non-metastasis

^b^*m* metastasis

^c^*DSH* domestic shorthair

^d^*N/D* Not determined

### Serum peptidomics profile results

**Fig. 1:**
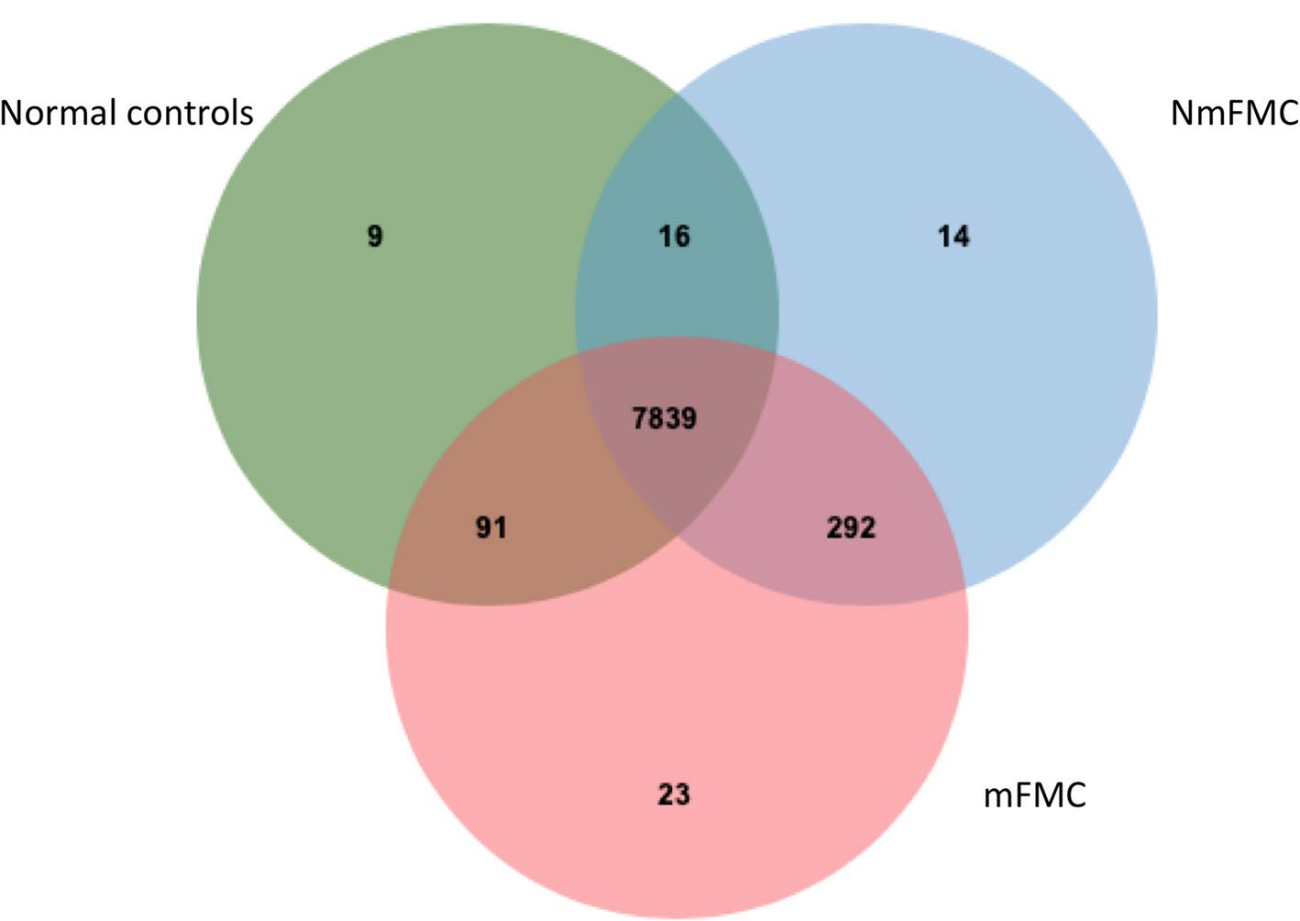
Venn diagram of proteins differentially expressed in NmFMC and mFMC and normal controls

Both peptides degraded from proteins and endogenous peptides were subject to analysis. However, it was observed that only peptides degraded from proteins exhibited differential expression. Out of a total of 8284 detected proteins, 14 were exclusively expressed in NmFMC, 23 in mFMC and 9 in the controls, as illustrated in the Venn diagram (Tables 2, 3 and 4; Fig. 1). Proteins uniquely observed in NmFMC included the double-stranded RNA-binding protein Staufen homolog 2 (STAU2), WW domain binding protein 11 (WBP11), proline and serine-rich coiled-coil 1 (PSRC1), complement C1q like 3 (C1QL3), fibroblast growth factor 14 (FGF14), BAZ2A and GABRE. Proteins solely identified in mFMC included B-cell lymphoma 6 transcription repressor (BCL6), thioredoxin reductase 3 (TXNRD3), ceruloplasmin (CP), baculoviral IAP repeat-containing 6 (BIRC6), POU class 5 homeobox 1 (POU5F1, also known as OCT4), laminin subunit alpha 1 (LAMA1), listerin E3 ubiquitin protein ligase 1 (LTN1), 1,4-alpha-glucan branching enzyme 1 (GBE1), calcium voltage-gated channel subunit alpha1 E (CACNA1E) and pleckstrin homology domain-containing S1 (PLEKHS1).

**Table 2:** Nominated proteins uniquely found in non-metastasis of feline mammary carcinoma based on molecular function by UniProtKB/Swiss-Prot

Database	Protein names	Peptides	Average protein expression ± SD	Molecular function
A0A5F5XG56	WW domain binding protein 11 (WBP11)	ELTPLQAMMLRMAGQEIPEEGR	3.35 ± 6.37	RNA processing
M3WM94	Proline and serine rich coiled-coil 1	LPVPSAIPRPASRMPLTSR	2.32 ± 5.67	Cytoplasm, cytoskeleton
A0A2I2V4V0	Bromodomain adjacent to zinc finger domain 2A (BAZ2A)	EEVAKGK	1.42 ± 5.13	DNA binding, metal ion binding
M3WMX9	Cysteine-rich PDZ-binding protein (CRIPT)	FSTCRICK	1.30 ± 4.69	microtubule binding, PDZ domain binding, protein-containing complex binding
M3W4A9	Gamma-aminobutyric acid type A receptor subunit epsilon (GABRE)	HPDIHARALMPPR	1.25 ± 4.49	GABA-A receptor activity, GABA-gated chloride ion channel activity, inhibitory extracellular ligand-gated ion channel activity, neurotransmitter receptor activity, transmitter-gated ion channel activity involved in regulation of postsynaptic membrane potential
A0A5F5XTH3	Family with sequence similarity 120B (FAM120B)	APGTAGQAKDSTGGIR	1.23 ± 4.43	
A0A5F5XHP5	Uncharacterized protein	AVGAN	1.20 ± 4.32	
A0A5F5Y0W5	Cell cycle progression 1 (CCPG1)	DQNVKQETDGK	1.20 ± 4.32	positive regulation of cell cycle, positive regulation of cell population proliferation, positive regulation of transcription by RNA polymerase II, regulation of Rho guanyl-nucleotide exchange factor activity
M3X401	Olfactory receptor (LOC101088169)	AFSTCASHFLVVSLFYGSVMVMYVSPGSRSHPGTQK	1.19 ± 4.30	G protein-coupled receptor activity, olfactory receptor activity
A0A2I2UAL5	Double-stranded RNA-binding protein Staufen homolog 2 (STAU2)	AQQAVANK	1.19 ± 4.30	double-stranded RNA binding
A0A337S2I6	Formin homology 2 domain containing 3 (FHOD3)	FNSGDLGR	1.18 ± 4.25	actin filament binding
A0A5F5Y181	Fibroblast growth factor (FGF14)	AAAIASGLIR	1.15 ± 4.14	growth factor activity
A0A2I2UTW8	von Willebrand factor A domain-containing protein 9 (INTS14)	PTVVVMDVSLSMTRPVSIEGSEEYQRK	1.11 ± 3.99	integrator complex activity
A0A337STI2	Complement C1q like 3 (C1QL3)	FTCSIPGIYFFTYHVLMRGGDGTSMWADLCK	1.04 ± 3.76	identical protein binding

**Table 3:** Nominated proteins uniquely found in metastasis of feline mammary carcinoma based on molecular function by UniProtKB/Swiss-Prot

Database	Protein names	Peptides	Average protein expression ± SD	Molecular function
M3W9Z2	Laminin subunit alpha 1 (LAMA1)	LAGALDSGLGSVR	3.14 ± 7.04	extracellular matrix structural constituent, glycosphingolipid binding, protein C-terminus binding, signaling receptor binding
A0A5F5XM44	Voltage-dependent R-type calcium channel subunit alpha (CACNA1E)	IHYTEMYEMLTLMSPPLGLGK	3.02 ± 5.91	calcium ion binding, voltage-gated calcium channel activity
A0A2I2UP49	FRAS1 related extracellular matrix 2 (FREM2)	GASTLRTLATGHLGFMITSK	2.66 ± 5.94	basement membrane, integral component of membrane, cell adhesion, cell communication, embryonic digit morphogenesis development, morphogenesis of an epithelium
A0A5F5XYI6	Cyclic AMP-responsive element-binding protein 3-like protein 2 (CREB3L2)	EYMDSLEKK	2.52 ± 5.63	cAMP response element binding, DNA-binding transcription activator activity, RNA polymerase II-specific
A0A337SWR8	Folliculin interacting protein 1 (FNIP1)	CSSDANMLGEMMFGSVAMSYK	2.41 ± 6.35	lysosomal membrane
A0A2I2UKP7	Interleukin 31 (IL31)	IILELRPMSKGLLQDYVSK	2.15 ± 5.67	cytokine activity, cytokine receptor binding
D3U664	POU domain protein (POU5F1)	FEALQLSFK	2.03 ± 5.38	chromatin DNA binding, cytokine binding, DNA-binding transcription activator activity, RNA polymerase II-specific, DNA-binding transcription factor activity, RNA polymerase II-specific, RNA polymerase II cis-regulatory region sequence-specific DNA binding, RNA polymerase II intronic transcription regulatory region sequence-specific DNA binding, transcription factor binding, ubiquitin protein ligase binding
A0A5F5Y685	Retinol dehydrogenase 11 (RDH11)	LTLSGPVLACRLSLVIPQACR	2.03 ± 5.37	aldehyde dehydrogenase (NADP +) activity
M3VVR7	Thioredoxin-disulfide reductase (TXNRD3)	LLGLIEGNR	1.80 ± 4.77	electron transfer activity, flavin adenine dinucleotide binding, protein disulfide oxidoreductase activity, thioredoxin-disulfide reductase activity
M3WID6	Glutamate receptor (GRIN2B)	TAKNMANLSGVNGSPQSALDFIR	1.78 ± 5.9	glutamate-gated calcium ion channel activity, glycine binding, ligand-gated ion channel activity, NMDA glutamate receptor activity, signaling receptor activity
A0A337SR21	1,4-alpha-glucan branching enzyme (GBE1)	GIQLHKMIR	1.44 ± 4.83	1,4-alpha-glucan branching enzyme activity, cation binding, hydrolase activity, hydrolyzing O-glycosyl compounds
M3WY11	Pleckstrin homology domain containing S1 (PLEKHS1)	GQHQRTGESHAR	1.41 ± 4.74	
M3WXJ0	Homer scaffold protein 2 (HOMER2)	TDIEESK	1.38 ± 4.57	actin binding, G protein-coupled glutamate receptor binding, synaptic receptor adaptor activity
M3W539	Baculoviral IAP repeat containing 6 (BIRC6)	AGKIFSQMNNIMSK	1.30 ± 4.29	cysteine-type endopeptidase inhibitor activity, ubiquitin conjugating enzyme activity
A0A5F5XXV1	UBX domain protein 2A (UBXN2A)	FNISHR	1.28 ± 4.24	acetylcholine receptor binding
A0A5F5XK47	Uncharacterized protein	ELPSPQSHPDTALLGQSPGAR	1.25 ± 4.29	
A0A337S289	EEF1A lysine methyltransferase 1 (EC 2.1.1.-) (EEF1AKMT1 N6AMT2)	EDFSIYIFEYDK	0.80 ± 3.84	nucleic acid binding, protein-lysine N-methyltransferase activity
A0A2I2UHD6	E3 ubiquitin-protein ligase listerin (LTN1)	VKDAAEGGSGSEEAGGR	0.71 ± 3.40	ribosomal large subunit binding, ubiquitin protein ligase activity, zinc ion binding
M3VZY5	Ceruloplasmin (CP)	VESIQCFQNTEAGSPTIMLLSLK	0.70 ± 3.37	ATP binding, hydrolase activity, acting on acid anhydrides, in phosphorus-containing anhydrides, nucleic acid binding, nucleosome-dependent ATPase activity, zinc ion binding
A0A291NGU8	IgH variable region (Fragment)	YWGQGALVTVSSASPK	0.70 ± 3.37	
A0A337SN80	BCL6 transcription repressor (BCL6)	DEFLNSRMLMPQDIMAYR	0.68 ± 3.26	DNA-binding transcription repressor activity, RNA polymerase II-specific, RNA polymerase II cis-regulatory region sequence-specific DNA binding
A0A337SQW1	Family with sequence similarity 189 member B (FAM189B)	MPSPSDSSR	0.65 ± 3.12	WW domain binding
M3W513	Polypeptide N-acetylgalactosaminyl-transferase (GALNT8)	IFLGVIGSLDGGMLVYGGENVELSLR	0.54 ± 2.6	carbohydrate binding, polypeptide N-acetylgalactosaminyltransferase activity

**Table 4:** Nominated proteins uniquely found in normal controls based on molecular function by UniProtKB/Swiss-Prot

Database	Protein names	Peptides	Average protein expression ± SD	Molecular function
A0A337RXV1	Zinc finger protein 26 (ZNF84)	THTGEKPHGCIQCGK	2.60 ± 5.78	DNA-binding transcription factor activity, RNA polymerase II-specific, metal ion binding, RNA polymerase II cis-regulatory region sequence-specific DNA binding
A0A337SAK8	Olfactory receptor (LOC101089999)	IPSAEGKQK	2.55 ± 5.70	G protein-coupled receptor activity, olfactory receptor activity
A0A5F5Y4V9	Transcription elongation factor A2 (TCEA2)	LLDASDAK	1.82 ± 5.14	nucleic acid binding, zinc ion binding
M3VZ25	Solute carrier family 27 member 3 (SLC27A3)	GHKVR	1.80 ± 5.09	long-chain fatty acid transporter activity, nucleotide binding
A0A337SXC3	Rap guanine nucleotide exchange factor 4 (RAPGEF4)	LGSGEGLIIVKMSSGGEK	1.80 ± 5.09	guanyl-nucleotide exchange factor activity
A0A5F5Y2R7	Scavenger receptor cysteine-rich domain-containing protein SCART1-like (SCART1)	GTEPTIRNCR	1.69 ± 4.76	scavenger receptor activity
M3VV17	Tubulin alpha chain (TUBAL3)	VGINSQPPTVTPGGDLAK	0.91 ± 3.74	GTPase activity, GTP binding, structural constituent of cytoskeleton
A0A5F5Y6C1	Histone methyltransferase SMYD2 (SMYD2)	HYPLYSLNVASMWLKLGR	0.89 ± 3.66	histone-lysine N-methyltransferase activity
A0A2I2U173	Serine/threonine-protein phosphatase 4 regulatory subunit 1-like (LOC101092674)	SNFPGVLADYLTPIVVRYLTDPNNQVR	0.78 ± 3.23

Furthermore, 42 proteins in NmFMC and mFMC exhibited at least twofold differential expression when compared with each other (*p* < 0.01) (Tables 5 and 6). Proteins significantly expressed in NmFMC compared with mFMC included coagulation factor XIII A chain (F13A1), centromere protein F (CENPF), pyruvate dehydrogenase phosphatase catalytic subunit 2 (PDP2), erythrocyte membrane protein band 4.1 (EPB41 or 4.1R), sorting nexin 10 (SNX10), galactosidase beta 1-like 2 (GLB1L2) and trafficking kinesin protein 2 (TRAK2). On the other hand, proteins highly expressed in mFMC compared with NmFMC included WD repeat domain 1 (WDR1), adenylate cyclase 10 (ADCY10) and activity-dependent neuroprotector homeobox (ADNP) (Supplementary Fig. 1).

**Table 5:** Overexpressed proteins with at least twofold differences of non-metastasis (NmFMC) compared with metastasis of feline mammary carcinoma (mFMC) and controls

Database	Protein names	Peptides	NmFMC		mFMC		Controls		Molecular function
			Median	IQR^d^	Median	IQR	Median	IQR	
A0A5F5XY49	Family with sequence similarity 246 member B (FAM246B)	SVYGASEALR	17.53	2.33	15.50	2.73	17.71	3.15	
A0A5F5XNK1	Katanin p60 ATPase-containing subunit a-like 2 (KATNAL2)	LLKPLSAFIGMNSEMR	16.16	0.92	14.12	15.9	13.78	16.40	ATP binding, isomerase activity, microtubule binding, microtubule-severing ATPase activity
M3W5A2	Trafficking kinesin protein 2 (TRAK2)	TPNAQENGR	17.37	1.58	15.78	2.89	17.48	2.70	GABA receptor binding, myosin binding, signaling receptor binding
M3W5L0	Pyruvate dehyrogenase phosphatase catalytic subunit 2 (PDP2)	EALMYSFQR	15.36	3.01	13.42	2.19	14.91	4.25	pyruvate dehydrogenase phosphatase activity, metal ion binding, protein serine/threonine phosphatase activity
A0A5F5XU84	Centromere protein F (CENPF)	AATQMLEELK	16.57	4.46	0.00	15.36	7.92	18.10	dynein complex binding, microtubule binding, protein homodimerization activity, transcription factor binding
M3W4Q9	Intraflagellar transport 22 (IFT22)	SSASGRAPADR	16.85	1.80	14.89	15.71	14.56	15.72	
M3WSU5	Methyl-CPG binding domain protein 5 (MBD5)	MFLSVSLQK	15.03	0.81	15.12	0.79	15.34	0.84	
M3VYG2	Cub and sushi multiple domains 2 (CSMD2)	ARMCDAHLR	15.07	0.75	13.85	14.93	15.45	2.16	membrane integral component
A0A337S9A6	Insulin receptor substrate 2 (IRS2)	AGAPK	15.00	1.03	14.68	0.78	14.74	0.91	insulin receptor binding, phosphatidylinositol 3-kinase binding, protein kinase binding
A0A5F5XXD4	Zinc finger protein 324 (ZNF324)	MATAALTDR	17.55	1.33	0.00	15.36	17.58	14.34	metal ion binding
A0A337S8X8	Uncharacterized protein	GPGMDVSGPK	14.88	1.05	13.93	1.37	14.66	1.38	
A0A5F5XL47	Rwd domain-containing protein 3 (RWDD3)	IILILLQGDR	16.94	2.88	0.00	14.94	0.00	19.14	positive regulation of protein SUMOylation
A0A5F5XCD6	Traf3 interacting protein 1 (TRAF3IP1)	AELAELEQLIRDQQDK	14.66	1.12	13.98	2.33	14.38	15.07	microtubule binding
A0A337S420	Band 4.1 (erythrocyte membrane protein band 4.1) (protein 4.1) (EPB41)	LTSTDTIPK	15.49	1.42	13.78	2.04	15.05	2.17	actin binding, calmodulin binding, structural molecule activity
A0A5F5Y758	Translocase of inner mitochondrial membrane domain containing 1 (TIMMDC1)	VFAAGAVAADSENQK	15.42	16.84	0.00	13.43	14.22	16.74	
A0A337SRT0	Neuronal-specific septin-3 (SEPTIN3)	SPGPAGPGSVGQK	14.87	18.78	0.00	0.00	6.83	16.47	GTP binding
A0A5F5XNQ6	Pparg related coactivator 1 (PPRC1)	WGQSPPPQQR	14.71	19.67	0.00	0.00	0.00	16.96	RNA binding, transcription coregulator activity, transcription factor binding
M3WL91	Collagen type v alpha 2 chain (COL5A2)	GDPGSHGRVGDR	14.12	14.54	0.00	10.24	0.00	13.76	extracellular matrix structural constituent, metal ion binding
M3WJD9	Beta-galactosidase-1-like protein 3 (GLB1L2)	NAEDVEDTVSK	13.38	17.65	0.00	0.00	0.00	14.57	beta-galactosidase activity
A0A5F5XWE8	Coagulation factor XIII A chain (F13A1)	EVGGDGIR	15.69	16.35	0.00	0.00	0.00	0.00	metal ion binding, protein-glutamine gamma-glutamyltransferase activity
A0A337SJJ4	Sorting nexin 10 (SNX10)	EEFVSVWVR	0.00	14.47	0.00	0.00	0.00	0.00	1-phosphatidylinositol binding, ATPase binding

denote a significant difference in the same row at *p* < 0.01

^d^*IQR* interquartile range

**Table 6:** Overexpressed proteins with at least twofold differences of metastasis (mFMC) compared with non-metastasis of feline mammary carcinoma (NmFMC) and controls

Database	Protein names	Peptides	mFMC		NmFMC		Controls	
			Median	IQR^d^	Median	IQR	Median	IQR
A0A2I2UY44	Phosphofurin acidic cluster sorting protein 1	DLNSVVIAVK	17.35	2.31	15.25	2.19	15.91	3.52
M3WWL3	Cytochrome P450 2F5 (CYP2F1)	DLIARSVR	15.16	1.25	14.26	1.82	0.00	15.53
A0A5F5XI19	ST3 beta-galactoside alpha-2,3-sialyltransferase 6 (ST3GAL6)	GGGSSLMEGDAK	16.92	2.88	13.93	15.48	14.20	16.18
A0A5F5Y4G3	L-2-hydroxyglutarate dehydrogenase (L2HGDH)	AQALDRDGNLIEDFVFDGGVGDIGNR	15.84	1.64	13.89	5.62	15.27	2.28
M3W363	ADAM metallopeptidase with thrombospondin type 1 motif 3 (ADAMTS3)	GTFTRTPR	14.38	1.17	13.37	13.74	14.56	2.41
A0A337S4J8	Secretoglobin family 3A member 1 (SCGB3A1)	SLLGSLMYLG	14.60	1.36	13.75	14.06	14.64	11.77
M3WEW2	WD repeat domain 1 (WDR1)	YTNLTLR	14.94	0.81	13.94	14.78	14.58	1.30
A0A2I2V2S6	Follistatin like 5 (FSTL5)	GNNCK	14.98	0.74	14.25	14.31	13.98	15.04
M3XE38	Activity dependent neuroprotector homeobox (ADNP)	DCEKYKPGVLLGFNMK	13.46	1.13	12.25	1.50	13.67	1.79
M3WFB1	Membrane bound transcription factor peptidase, site 1 (MBTPS1 SLC38A8)	RVLWDQYHNLR	13.98	1.39	10.17	12.63	0.00	0.00
M3WLU1	Adenylate cyclase 10 (ADCY10)	ISFHQNFYTIQIFMATVLGLNTCKHYK	13.09	1.52	11.30	12.29	12.23	13.23
A0A2I2UCH4	Transmembrane protein 255B (TMEM255B)	MQPPVPGPLALLDNTEGFARR	13.94	4.41	0.00	12.00	0.00	9.27
A0A337SDK0	Rho GTPase activating protein 21 (ARHGAP21)	GNEAYSGNAR	14.06	1.83	0.00	14.43	11.56	14.65
M3WW40	Pre-mRNA processing factor 39 (PRPF39)	RHGNMEEAEHLLQDAIK	14.19	9.17	0.00	0.00	14.94	13.49
M3WS25	Caspase-14-like (LOC101084312)	DGERVSLEDIFEMFNNK	14.03	2.93	11.92	13.01	12.02	14.87
M3WU27	Coiled-coil domain containing 73 (CCDC73)	EKEIEGLK	14.20	1.71	0.00	12.63	14.26	11.64
A0A337SU95	Testis associated actin remodelling kinase 2 (TESK2)	VREIPPFR	14.15	10.69	0.00	0.00	0.00	14.40
M3W938	Ganglioside induced differentiation associated protein 1 like 1 (GDAP1L1)	RHLANATTDLMK	12.88	1.84	0.00	12.25	10.31	13.69
M3W3G6	[Heparan sulfate]-glucosamine N-sulfotransferase (EC 2.8.2.8) (NDST3)	EGTRMNTNDVK	12.55	2.52	0.00	11.36	0.00	7.77
M3WJL7	Methyltransferase like 3 (METTL3)	LSAMMGAVAEK	13.09	9.51	0.00	0.00	0.00	8.86
M3WUK1	Uroplakin 2 (UPK2)	KMESIGLGMAR	15.46	16.73	0.00	0.00	0.00	17.60

denote a significant difference in the same row at *p* < 0.01

^d^*IQR* interquartile range

**Fig. 2:**
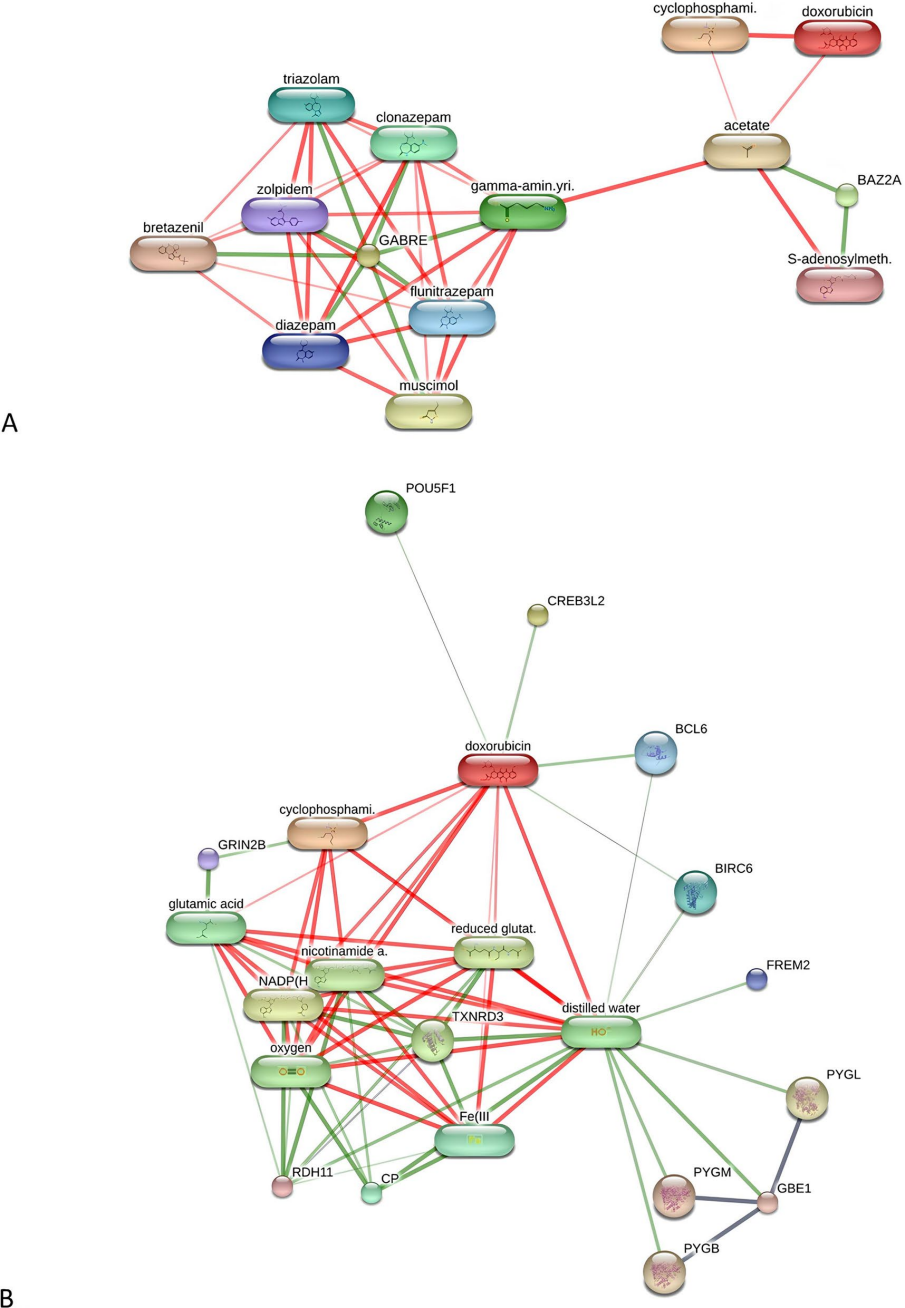
Involvement of serum proteins in FMC and chemotherapy drugs, doxorubicin and cyclophosphamide, in networks of protein − chemotherapy drug interactions. **A** Serum proteins in NmFMC include bromodomain adjacent to zinc finger domain 2A (BAZ2A) and gamma-aminobutyric acid type A receptor subunit epsilon (GABRE). **B** Serum proteins in mFMC include POU domain protein (POU5F1), cyclic AMP-responsive element-binding protein 3-like protein 2 (CREB3L2), BCL6 transcription repressor (BCL6), baculoviral IAP repeat containing 6 (BIRC6), FRAS1 related extracellular matrix 2 (FREM2), 1,4-alpha-glucan branching enzyme (GBE1), glutamate receptor (GRIN2B), thioredoxin-disulfide reductase (TXNRD3), ceruloplasmin (CP) and retinol dehydrogenase 11 (RDH11)

In addition, 280 proteins in NmFMC, 616 proteins in mFMC and 170 proteins commonly found in both NmFMC and mFMC were differently expressed compared with the controls (*p* < 0.01). Among these, insulin receptor (INSR), SR-related CTD associated factor 1 (SCAF1) and pyruvate dehydrogenase kinase 1 (PDK1) were differentially expressed in NmFMC compared with controls, whereas ligand-dependent nuclear receptor corepressor (LCOR) was differentially expressed in mFMC compared with controls. In addition, endoglin (ENG), checkpoint kinase 1 (CHEK1), epidermal growth factor receptor (EGFR) and DEAH-box helicase 32 (putative) (DHX32) were significantly expressed in both NmFMC and mFMC compared with controls (Supplementary Tables 1 − 3). Moreover, regarding the relationship with chemotherapy drugs, either doxorubicin or cyclophosphamide, associations were observed for some proteins found exclusively in the NmFMC group, including GABRE and BAZ2A, and in the mFMC group, including BCL6, TXNRD3, BIRC6 and GBE1 (Fig. 2) [30]. However, no associations with chemotherapy drugs were exhibited in nine proteins uniquely expressed in a control group.

## Discussion

The present study sheds light on the differential protein expression observed in NmFMC and mFMC at the peptidome level. Notably, both pro- (e.g., STAU2, BAZ2A and GABRE) and anti-proliferative proteins (e.g., C1QL3 and EPB41) were identified in NmFMC, while proteins associated with poor prognosis (e.g., BCL6, TXNRD3 and CP) and metastasis (e.g., POU5F1 and LAMA1) were prominent in the mFMC group. The upregulation of STAU2, observed in T and B cells in human breast cancer patients may promote tumor growth through the RNA transport process of various inflammatory cytokine molecules suggesting its potential as a novel diagnostic biomarker for human breast cancer screening [31]. Similarly, WBP11 has been linked to the activation of the fibroblast growth factor receptor (FGFR)-Wingless/Integrated (Wnt)-β-catenin pathway in human gastric cancer [32]. Additionally, PSRC1 implicated in cancer cell proliferation and was downregulated by the tumor suppressor p53 in human hepatocellular carcinoma [33]. Moreover, several candidates identified in the NmFMC group, including BAZ2A, GABRE, INSR, SCAF1, PDK1 and PDP2, have been proposed as potential therapeutic targets. BAZ2A and GABRE, uniquely expressed in NmFMC, exhibited relationship with chemotherapy drugs (Fig. 2A), suggesting their significance in treatment response [11, 12]. In human triple-negative breast cancer, inhibition of BAZ2A has been demonstrated to induce apoptosis, while BAZ2A has also been implicated in regulating hypermethylation, contributing to advanced tumor stages and recurrence in prostate cancer [34]. GABRE activation has the potential to sensitize cancer cells to radiation, chemotherapeutic agents and immune checkpoint inhibitors [12].

Remarkably, proteins prominently expressed in NmFMC compared with other groups, such as F13A1, CENPF, INSR, SCAF1 and PDK1, have been implicated in human breast cancer, further supporting the potential use of FMC as a model for studying human breast cancer. For instance, F13A1 was prominently expressed in human estrogen receptor-negative breast cancer, while targeting CENPF resulted in tumor growth inhibition in human breast cancer [35, 36]. Differential splicing of INSR occurs more commonly in human breast cancer than in non-tumor breast tissues, and *SCAF1* has been proposed as a cancer prognostic biomarker [37, 38]. Furthermore, PDK1 plays a role in the growth and survival of human breast cancer cells [39, 40]. Paradoxically, a group of proteins, including C1QL3, EPB41, SNX10, FGF14, GLB1L2 and TRAK2, have been reported as good prognostic markers or tumor suppressors in the NmFMC group. Notably, complement C1q was previously shown to be associated with extended disease-free survival in basal-like breast cancer and improved overall survival in HER2-positive breast cancer in humans [41]. Elevated levels of EPB41 expression have been correlated with prolong survival in human breast cancer patients [42]. Moreover, FGF14 and SNX10 have demonstrated tumor suppressive properties in colorectal cancer [43, 44]. Conversely, decreased expression of GLB1L2 and TRAK2 has been documented in prostate cancer and osteosarcoma, respectively [23, 45]. Hence, these proteins have the potential to serve as good prognostic biomarkers for FMC, especially NmFMC. The coexistence of both pro- and anti-proliferative proteins, acting as tumor promoters and suppressors, respectively, presents a distinctive characteristic of NmFMC. A comprehensive examination of their protein expression, with a particular focus on its correlation with survival outcomes, necessitates further investigation in a larger patient cohort.

In the mFMC group, there was marked protein expression of BCL6, TXNRD3, CP and BIRC6, which have been linked with poor prognosis in human breast cancer [46–50]. BCL6, identified as a master transcription factor for regulating follicular helper cell proliferation, has been demonstrated to inhibit apoptosis, thereby promoting tumor invasion, migration and growth. BCL6 expression also promotes tumor angiogenesis and is associated with human breast cancer progression and poor prognosis [47]. Moreover, BCL6 inhibitors have shown potent effects against these tumor types [47, 51]. TXNRD3 is involved in oxidative stress and has been associated with poor prognosis in various cancers [48]. CP is a plasma protein for copper binding and is associated with various immune pathways and inflammatory responses related to the tumor microenvironment. In invasive human breast cancer, low levels of this protein were correlated with low tumor immune cell infiltration status and better prognosis [49]. BIRC6 has demonstrated overexpression in triple-negative human breast cancer cells and tissues, positively correlated with epidermal growth factor receptor (EGFR), and associated with poor patient survival time [50].

Proteins significantly upregulated in mFMC compared to NmFMC included WDR1, which is associated with cell motility. Overexpression of this protein correlated with shorter distant metastasis-free survival, especially in basal-like tumors of human breast cancer [52]. Notably, unique metastatic biomarkers found in mFMC, such as POU5F1 (OCT4) and LAMA1, have been identified in human breast cancer [53, 54]. POU5F1 has been reported as a biomarker in both undifferentiated cells and several cancer cells, suggesting shared characteristics between these cell types. A previous study identified POU5F1 as a potential candidate for predicting metastasis in human breast cancer [53]. LAMA1 has been shown to mediate cell attachment, migration and tissue organization. In metastatic human breast tumors, overexpression of fibronectin and LAMA1 proteins were exhibited in mice, promoting the degradation processes of extracellular matrix proteins in cancer metastasis [54]. Moreover, several proteins prominently observed in mFMC in this study have been reported as potential poor prognostic markers in various other cancers. These proteins include LTN1 (ovarian cancer), GBE1, CACNA1E and ADCY10 (lung cancers), as well as PLEKHS1 and ADNP (bladder cancer) [55–59]. Additionally, another group of proteins notably found in both NmFMC and mFMC compared to the controls (*p* < 0.01) consisted of ENG, CHEK1, EGFR and DHX32. All of these proteins have been associated with poor or unfavorable prognosis in human breast cancer [60–63]. Inhibition of ENG has been shown to prevent tumor angiogenesis and metastatic spread in human breast cancer [60]. High expression of CHEK1 in Nigerian human breast cancer patients is associated with an aggressive phenotype and poor prognosis [61]. EGFR has been linked to the pathogenesis and progression of human breast cancer [62–64]. DHX32 expression has been associated with a poor prognosis in human breast cancer patients [63]. Several proteins found in the present study, including EGFR, BIRC6 and FGF, are associated with EGF. The functions of these proteins and their association with novel FMC diagnostic and/or prognostic biomarkers should be further investigated. The limitations of the present study include a restricted population size, the absence of tissue proteomics profiles and a lack of long-term follow-up data due to infrequent return visits by most cat patients after surgery. Further research involving a larger population and a comparison with tissue proteomics profiles is necessary to investigate the precise roles of these candidates.

## Conclusion

Serum peptidomics revealed potential candidates that were either uniquely or highly expressed in NmFMC and mFMC. In NmFMC, diagnostic candidates with paradoxical characteristics were observed, displaying either the promotion or suppression of cell proliferation, highlighting the distinctive nature of this type of cancer. Meanwhile, potential poor prognostic and metastatic candidates were identified in mFMC. The relationship of proteins in NmFMC or mFMC with chemotherapy drugs was observed. The discovery of similar protein candidates in both FMC and human breast cancer supports the potential utility of FMC as a model for studying mechanisms and identifying therapeutic targets in human breast cancer.

## Materials and methods

### Animals

A cross-sectional study was conducted involving 13 cats diagnosed with spontaneous NmFMC, 23 with spontaneous mFMC and 18 healthy cats. Initially, patients were staged according to the TNM system: stage I (tumor diameter < 2 cm), stage II (tumor diameter 2 to 3 cm), stage III (tumor diameter < 3 cm with lymph node metastasis or tumor diameter > 3 cm) or stage IV (any tumor size with lymph node or distant metastasis). Staging was confirmed by histopathology, indicating the presence of FMC [5]. The patients were categorized into 13 samples with NmFMC, characterized by the absence of lymph node or distant metastases, and 23 samples with mFMC, demonstrating lymph node and/or distant metastases (Table 1). Thoracic radiographs, including ventrodorsal and lateral views, were examined to identify distant metastases. Whole blood samples from a control group were collected from 18 healthy cats visiting the Small Animal Hospital, Faculty of Veterinary Science, Chulalongkorn University, with no history or clinical signs of mammary disease. The study was conducted following the ethical guidelines required by the Chulalongkorn University Animal Care and Use Committee (CU-ACUC), Thailand (approval number 1831091) and written informed consents were obtained from all cat owners.

### Sample collection and preparation

Whole blood samples were collected once from the cephalic or saphenous veins of both patients before surgery and from a control group. After collection, samples were centrifuged at 3000 × g for 15 min at 4 °C to obtain serum. The serum was then mixed with Halt protease inhibitor cocktail (Thermo Fisher Scientific, Waltham, MA, USA) and stored at –20 °C until analysis.

### LC–MS/MS analysis and data processing

Total protein concentrations were assessed using the colorimetric Pierce Modified Lowry’s assay (Thermo Fisher Scientific, Waltham, MA, USA), based on the reduction of Folin-Ciocalteu reagent by Tyr and Trp residues in proteins under alkaline conditions. Protein samples at 0.1 μg/μL in 0.1% formic acid were processed using Nanosep Centrifugal Devices with a 10 K Omega membrane (Pall Corporation, Port Washington, NY, USA) to remove proteins larger than 10 kDa. Peptide separation was performed using A 75 μm diameter × 5 cm length Acclaim PepMap nanocolumn (Thermo Fisher Scientific). The nanoLC system was connected to electrospray ionization MS in positive ion mode and quadrupole ion-trap MS (Bruker Daltonics, Billerica, MA, USA). Peptides were eluted with a 4–70% linear gradient of eluent B (80% acetonitrile in water containing 0.1% formic acid) at a flow rate of 0.3 μL/min for 20 min. Regeneration and equilibration were carried out with 90% and 4% eluent B, respectively, for 40 min per run. A scan range of 400–1500 m/z, 3 averages, and up to 5 precursor ions selected from the MS scan at 200–2800 m/z were used for peptide fragment mass spectra analysis in data-dependent AutoMS mode. The LC–MS/MS results were converted into an mzXML file using CompassXport software (Bruker Daltonics). Protein quantification was performed based on peptide intensity using DeCyder MS Differential Analysis software (GE Healthcare, Chicago, IL, USA). PepDetect in MS mode facilitated automated peptide detection, charge state assignments, and assessment of peptide ion signal intensities. Proteins were identified based on one or more peptides with a MASCOT score corresponding to *p* < 0.05 (Matrix Science, Boston, MA, USA) and were annotated using the NCBI *Felis catus* database. The false discovery rate (FDR) was analyzed using Metaboanalyst 5.0 software, and low confidence identifications were removed [65]. Protein sequences and molecular functions were annotated using UniProtKB/Swiss-Prot entries (http://www.uniprot.org/). The relationship between sample groups was visualized using a jVenn diagram [66]. The association between candidate proteins and chemotherapy drugs was analyzed using Stitch version 5.0 [67]. The hierarchical abundance of nominated proteins in each group was represented using Morpheus heatmap (https://software.broadinstitute.org/morpheus).

### Statistical analysis

Differential protein expression in controls, NmFMC and mFMC was analyzed using the R package. Normality testing was conducted using the Shapiro–Wilk test, and statistical significance was determined using the Mann–Whitney U test in R, with a significant level set at *p* < 0.05.

### Supplementary Information

Additional file 1: Supplementary Table 1. Protein expression in non-metastatic feline mammary carcinoma (NmFMC) compared with controls.

Additional file 2: Supplementary Table 2. Protein expression in metastatic feline mammary carcinoma (mFMC) compared with controls.

Additional file 3: Supplementary Table 3. Protein expression in non-metastatic (NmFMC) and metastatic feline mammary carcinoma (mFMC) compared with controls.

Additional file 4: Supplementary Fig. 1. Partial least squares discriminant analysis (PLS-DA) plot depicting prominent proteins differentially expressed between non-metastatic (NmFMC) and metastatic feline mammary carcinoma (mFMC). (A) centromere protein F (CENPF). (B) erythrocyte membrane protein band 4.1 (EPB41). (C) trafficking kinesin protein 2 (TRAK2). (D) WD repeat domain 1(WDR1) (E) adenylate cyclase 10 (ADCY10). (F) activity-dependent neuroprotector homeobox (ADNP).

## Data Availability

The datasets generated and/or analysed during the current study are available in the ProteomeXchange repository, PXD035906.

## Abbreviations

ADCY10: Adenylate cyclase 10
ADNP: Activity-dependent neuroprotector homeobox
BAZ2A: Bromodomain adjacent to zinc finger domain 2A
BCL6: B-cell lymphoma 6 transcription repressor
BIRC6: Baculoviral IAP repeat containing 6
CACNA1E: Calcium voltage-gated channel subunit alpha1 E
CENPF: Centromere protein F
CHEK1: Checkpoint kinase 1
CP: Ceruloplasmin
CU-ACUC: Chulalongkorn University Animal Care and Use Committee
C1QL3: Complement C1q like 3
DHX32: DEAH-box helicase 32
EGFR: Epidermal growth factor receptor
ENG: Endoglin
EPB41: Erythrocyte membrane protein band 4.1
FGF14: Fibroblast growth factor 14
FMC: Feline mammary carcinoma
GABRE: Gamma-aminobutyric acid type A receptor subunit epsilon
GBE1: 1,4-Alpha-glucan branching enzyme 1
GLB1L2: Galactosidase beta 1-like 2
INSR: Insulin receptor
LAMA1: Laminin subunit alpha 1
LC: Liquid chromatography
LCOR: Ligand-dependent nuclear receptor corepressor
LTN1: Listerin E3 ubiquitin protein ligase 1
MS: Mass spectrometry
mFMC: Metastatic feline mammary carcinoms
NmFMC: Non-metastatic feline mammary carcinoma
PDK1: Pyruvate dehydrogenase kinase 1
PDP2: Pyruvate dehydrogenase phosphatase catalytic subunit 2
PLEKHS1: Pleckstrin homology domain containing S1
POU5F1: POU class 5 homeobox 1
PSRC1: Proline and serine rich coiled-coil 1
SCAF1: SR-related CTD associated factor 1
SNX10: Sorting nexin 10
STAU2: Double-stranded RNA-binding protein Staufen homolog 2
TNM: Tumor, node and metastasis
TRAK2: Trafficking kinesin protein 2
TXNRD3: Thioredoxin reductase 3
WBP11: WW domain binding protein 11
WDR1: WD repeat domain 1
WHO: World Health Organization

## References

[1] Sorenmo K, Worley DR, Goldschmidt MH. Small animal clinical oncology. In: Withrow M, editor. 5th ed. Missouri: Saunders Elsevier; 2013. p. 538–56.

[2] Giménez F, Hecht S, Craig LE, Legendre AM (2010). Early detection, aggressive therapy: optimizing the management of feline mammary masses. J Feline Med Surg.

[3] Zappulli V, Caliari D, Rasotto R, Ferro S, Castagnaro M, Goldschmidt M (2013). Proposed classification of the feline “complex” mammary tumors as ductal and intraductal papillary mammary tumors. Vet Pathol.

[4] de Campos CB, Damasceno KA, Gamba CO, Ribeiro AM, Machado CJ, Lavalle GE (2015). Evaluation of prognostic factors and survival rates in malignant feline mammary gland neoplasms. J Feline Med Surg.

[5] Seixas F, Palmeira C, Pires MA, Bento MJ, Lopes C (2011). Grade is an independent prognostic factor for feline mammary carcinomas: a clinicopathological and survival analysis. Vet J.

[6] McNeill C, Sorenmo K, Shofer F, Gibeon L, Durham A, Barber L (2009). Evaluation of adjuvant doxorubicin-based chemotherapy for the treatment of feline mammary carcinoma. J Vet Intern Med.

[7] Andrew NC (2003). Principles of treatment for mammary gland tumors. Clin Tech Small Anim Pract.

[8] Soares M, Correia J, Peleteiro MC, Ferreira F (2016). St Gallen molecular subtypes in feline mammary carcinoma and paired metastases—disease progression and clinical implications from a 3-year follow-up study. Tumor Biol.

[9] Soares M, Ribeiro R, Najmudin S, Gameiro A, Rodrigues R, Cardoso F (2016). Serum HER2 levels are increased in cats with mammary carcinomas and predict tissue HER2 status. Oncotarget.

[10] Marques CS, Soares M, Santos A, Correia J, Ferreira F (2017). Serum SDF-1 levels are a reliable diagnostic marker of feline mammary carcinoma, discriminating HER2-overexpressing tumors from other subtypes. Oncotarget.

[11] Nascimento C, Urbano AC, Gameiro A, Ferreira J, Correia J, Ferreira F (2020). Serum PD-1/PD-L1 levels, tumor expression and PD-L1 somatic mutations in HER2-positive and triple negative normal-like feline mammary carcinoma subtypes. Cancers (Basel).

[12] Bevill SM, Olivares-Quintero JF, Sciaky N, Golitz BT, Singh D, Beltran AS (2019). GSK2801, a BAZ2/BRD9 bromodomain inhibitor, synergizes with BET inhibitors to induce apoptosis in triple-negative breast cancer. Mol Cancer Res.

[13] Bhattacharya D, Gawali VS, Kallay L, Toukam DK, Koehler A, Stambrook P (2021). Therapeutically leveraging GABAA receptors in cancer. Exp Biol Med.

[14] Adega F, Borges A, Chaves R (2016). Cat mammary tumors: genetic models for the human counterpart. Vet Sci.

[15] Nascimento C, Ferreira F (2021). Tumor microenvironment of human breast cancer, and feline mammary carcinoma as a potential study model. Biochimica et Biophysica Acta (BBA) - Rev Can.

[16] Sommerville L, Howard J, Evans S, Kelly P, McCann A (2022). Comparative gene expression study highlights molecular similarities between triple negative breast cancer tumours and feline mammary carcinomas. Vet Comp Oncol.

[17] Zappulli V, Rasotto R, Caliari D, Mainenti M, Peña L, Goldschmidt MH (2015). Prognostic evaluation of feline mammary carcinomas. Vet Pathol.

[18] Soares M, Madeira S, Correia J, Peleteiro M, Cardoso F, Ferreira F (2016). Molecular based subtyping of feline mammary carcinomas and clinicopathological characterization. Breast.

[19] Greening DW, Simpson RJ. Characterization of the low-molecular-weight human plasma peptidome. In: Greening DW, Simpson RJ, editors. Serum/Plasma Proteomics: Methods and Protocols. New York, NY: Springer New York; 2017. p. 63–79. 10.1007/978-1-4939-7057-5_6 28674878

[20] Sukumolanan P, Phanakrop N, Thaisakun S, Roytrakul S, Petchdee S (2021). Analysis of the serum peptidomics profile for cats with sarcomeric gene mutation and hypertrophic cardiomyopathy. Front Vet Sci.

[21] Ploypetch S, Jaresitthikunchai J, Phaonakrop N, Sakcamduang W, Manee-in S, Suriyaphol P (2022). Utilizing MALDI-TOF MS and LC-MS/MS to access serum peptidome-based biomarkers in canine oral tumors. Sci Rep.

[22] Shen Y, Tolić N, Liu T, Zhao R, Petritis BO, Gritsenko MA (2010). Blood peptidome-degradome profile of breast cancer. PLoS ONE.

[23] Wang F, Ye B, Liu J, Kong D (2020). miR-487b and TRAK2 that form an axis to regulate the aggressiveness of osteosarcoma, are potential therapeutic targets and prognostic biomarkers. J Biochem Mol Toxicol.

[24] Marsilio S, Dröes FC, Dangott L, Chow B, Hill S, Ackermann M (2021). Characterization of the intestinal mucosal proteome in cats with inflammatory bowel disease and alimentary small cell lymphoma. J Vet Intern Med.

[25] Yu J, Boland L, Catt M, Puk L, Wong N, Krockenberger M (2023). Serum proteome profiles in cats with chronic enteropathies. J Vet Intern Med.

[26] Franco-Martínez L, Gelemanović A, Horvatić A, Contreras-Aguilar MD, Dąbrowski R, Mrljak V (2020). Changes in serum and salivary proteins in canine mammary tumors. Animals.

[27] Zamani-Ahmadmahmudi M, Nassiri SM, Rahbarghazi R (2014). Serological proteome analysis of dogs with breast cancer unveils common serum biomarkers with human counterparts. Electrophoresis.

[28] Ratcliffe L, Mian S, Slater K, King H, Napolitano M, Aucoin D (2009). Proteomic identification and profiling of canine lymphoma patients. Vet Comp Oncol.

[29] Zheng JS, Wei RY, Wang Z, Zhu TT, Ruan HR, Wei X (2020). Serum proteomics analysis of feline mammary carcinoma based on label-free and PRM techniques. J Vet Sci.

[30] Petrucci G, Henriques J, Gregório H, Vicente G, Prada J, Pires I (2021). Metastatic feline mammary cancer: prognostic factors, outcome and comparison of different treatment modalities – a retrospective multicentre study. J Feline Med Surg.

[31] Puttipanyalears C, Denariyakoon S, Angsuwatcharakon P, Aksornkitti V, Vongsaisuwan M, Asayut S (2021). Quantitative STAU2 measurement in lymphocytes for breast cancer risk assessment. Sci Rep.

[32] Wang L, Yu T, Li W, Li M, Zuo Q, Zou Q (2019). The miR-29c-KIAA1199 axis regulates gastric cancer migration by binding with WBP11 and PTP4A3. Oncogene.

[33] Hsieh WJ, Hsieh SC, Chen CC, Wang FF (2008). Human DDA3 is an oncoprotein down-regulated by p53 and DNA damage. Biochem Biophys Res Commun.

[34] Gu L, Frommel SC, Oakes CC, Simon R, Grupp K, Gerig CY (2015). BAZ2A (TIP5) is involved in epigenetic alterations in prostate cancer and its overexpression predicts disease recurrence. Nat Genet.

[35] Rezaul K, Thumar JK, Lundgren DH, Eng JK, Claffey KP, Wilson L (2010). Differential protein expression profiles in estrogen receptor-positive and -negative breast cancer tissues using label-free quantitative proteomics. Genes Cancer.

[36] Chen Q, Xu H, Zhu J, Feng K, Hu C (2020). LncRNA MCM3AP-AS1 promotes breast cancer progression via modulating miR-28-5p/CENPF axis. Biomed Pharmacother.

[37] Huang G, Song C, Wang N, Qin T, Sui S, Obr A (2020). RNA-binding protein CUGBP1 controls the differential INSR splicing in molecular subtypes of breast cancer cells and affects cell aggressiveness. Carcinogenesis.

[38] Adamopoulos PG, Raptis GD, Kontos CK, Scorilas A (2018). Discovery and expression analysis of novel transcripts of the human SR-related CTD-associated factor 1 (SCAF1) gene in human cancer cells using Next-Generation Sequencing. Gene.

[39] Kaplon J, Zheng L, Meissl K, Chaneton B, Selivanov VA, Mackay G (2013). A key role for mitochondrial gatekeeper pyruvate dehydrogenase in oncogene-induced senescence. Nature.

[40] Wang N, Fu J, Li Z, Jiang N, Chen Y, Peng J (2022). The landscape of PDK1 in breast cancer. Cancers (Basel).

[41] Mangogna A, Agostinis C, Bonazza D, Belmonte B, Zacchi P, Zito G (2019). Is thecomplement protein C1q a pro- or anti-tumorigenic factor? Bioinformatics analysis involving human carcinomas. Front Immunol.

[42] Feng  G, Guo  K, Yan Q, Ye  Y, Shen M, Ruan S (2019). Expression of protein 4.1 family in breast cancer: database mining for 4.1 family members in malignancies. Med Sci Monit.

[43] Ferguson HR, Smith MP, Francavilla C (2021). Fibroblast growth factor receptors (FGFRs) and noncanonical partners in cancer signaling. Cells.

[44] Zhang S, Yang Z, Bao W, Liu L, You Y, Wang X (2020). SNX10 (sorting nexin 10) inhibits colorectal cancer initiation and progression by controlling autophagic degradation of SRC. Autophagy.

[45] Kashanizadeh MG, Rezaei Fakhrnezhad F, Yavari S, Alizadeh H, Hashemim P, Monfaredan A (2021). Molecular expression of some oncogenes and predisposing behaviors contributing to the aggressiveness of prostate cancer. Rep Biochem Mol Biol.

[46] Tamma R, Ruggieri S, Annese T, Simone G, Mangia A, Rega S (2018). Bcl6/p53 expression, macrophages/mast cells infiltration and microvascular density in invasive breast carcinoma. Oncotarget.

[47] Yu JM, Sun W, Hua F, Xie J, Lin H, Zhou DD (2015). BCL6 induces EMT by promoting the ZEB1-mediated transcription repression of E-cadherin in breast cancer cells. Cancer Lett.

[48] Wu W, Li D, Feng X, Zhao F, Li C, Zheng S (2021). A pan-cancer study of selenoprotein genes as promising targets for cancer therapy. BMC Med Genomics.

[49] Chen F, Han B, Meng Y, Han Y, Liu B, Zhang B (2021). Ceruloplasmin correlates with immune infiltration and serves as a prognostic biomarker in breast cancer. Aging.

[50] Li Y, Tan Y, Wen L, Xing Z, Wang C, Zhang L (2021). Overexpression of BIRC6 driven by EGF-JNK-HECTD1 signaling is a potential therapeutic target for triple-negative breast cancer. Mol Ther Nucleic Acids.

[51] Cardenas MG, Oswald E, Yu W, Xue F, MacKerell AD, Melnick AM (2017). The expanding role of the BCL6 oncoprotein as a cancer therapeutic target. Clin Cancer Res.

[52] Lee JH, Kim JE, Kim BG, Han HH, Kang S, Cho NH (2016). STAT3-induced WDR1 overexpression promotes breast cancer cell migration. Cell Signal.

[53] Cai S, Geng S, Jin F, Liu J, Qu C, Chen B (2016). POU5F1/Oct-4 expression in breast cancer tissue is significantly associated with non-sentinel lymph node metastasis. BMC Cancer.

[54] Rocha M, Arcanjo R, Lopes C, Carneiro M, Souza A, Báo S (2017). Modulation of fibronectin and laminin expression by Rhodium (II) citrate-coated maghemite nanoparticles in mice bearing breast tumor. Sci Rep.

[55] He T, Huang L, Li J, Wang P, Zhang Z (2021). Potential prognostic immune biomarkers of overall survival in ovarian cancer through comprehensive bioinformatics analysis: a novel artificial intelligence survival prediction system. Front Med (Lausanne).

[56] Liang Y, Lei Y, Liang M, Du M, Liu Z, Li X (2022). GBE1 is an independent prognostic marker and associated with CD163(+) tumor-associated macrophage infiltration in lung adenocarcinoma. Front Oncol.

[57] Gao SH, Wang GZ, Wang LP, Feng L, Zhou YC, Yu XJ (2022). Mutations and clinical significance of calcium voltage-gated channel subunit alpha 1E (CACNA1E) in non-small cell lung cancer. Cell Calcium.

[58] Pignot G, Le Goux C, Vacher S, Schnitzler A, Radvanyi F, Allory Y (2019). PLEKHS1: A new molecular marker predicting risk of progression of non-muscle-invasive bladder cancer. Oncol Lett.

[59] Zhu S, Xu Z, Zeng Y, Long Y, Fan G, Ding Q (2020). ADNP upregulation promotes bladder cancer cell proliferation via the AKT pathway. Front Oncol.

[60] Paauwe M, Heijkants RC, Oudt CH, van Pelt GW, Cui C, Theuer CP (2016). Endoglin targeting inhibits tumor angiogenesis and metastatic spread in breast cancer. Oncogene.

[61] Ebili HO, Iyawe VO, Adeleke KR, Salami BA, Banjo AA, Nolan C (2018). Checkpoint kinase 1 expression predicts poor prognosis in Nigerian breast cancer patients. Mol Diagn Ther.

[62] Masuda H, Zhang D, Bartholomeusz C, Doihara H, Hortobagyi GN, Ueno NT (2012). Role of epidermal growth factor receptor in breast cancer. Breast Cancer Res Treat.

[63] Wang M, Zhang G, Wang Y, Ma R, Zhang L, Lv H (2017). DHX32 expression is an indicator of poor breast cancer prognosis. Oncol Lett.

[64] Lluch A, Eroles P, Perez-Fidalgo JA (2014). Emerging EGFR antagonists for breast cancer. Expert Opin Emerg Drugs.

[65] Pang Z, Chong J, Zhou  G, de Lima Morais DA, Chang  L, Barrette M (2021). MetaboAnalyst 5.0: narrowing the gap between raw spectra and functional insights. Nucleic Acids Res.

[66] Bardou P, Mariette J, Escudié F, Djemiel C, Klopp C (2014). jvenn: an interactive Venn diagram viewer. Venn diagram viewer BMC Bioinformatics.

[67] Szklarczyk D, Santos A, von Mering C, Jensen LJ, Bork P, Kuhn M (2016). STITCH 5: augmenting protein–chemical interaction networks with tissue and affinity data. Nucleic Acids Res.

