# Supplementary material for: Analysis of serum peptidome profiles of non-metastatic and metastatic feline mammary carcinoma using liquid chromatography-tandem mass spectrometry
Source: BMC Vet Res. 2024 Jun 29;20:280. doi: 10.1186/s12917-024-04148-y (PMC11218297; doi:10.1186/s12917-024-04148-y)
Supplement: Supplementary file 4 — Additional file 4: Supplementary Fig. 1. Partial least squares discriminant analysis (PLS-DA) plot depicting prominent proteins differentially expressed between non-metastatic (NmFMC) and metastatic feline mammary carcinoma (mFMC). (A) centromere protein F (CENPF). (B) erythrocyte membrane protein band 4.1 (EPB41). (C) trafficking kinesin protein 2 (TRAK2). (D) WD repeat domain 1(WDR1) (E) adenylate cyclase 10 (ADCY10). (F) activity-dependent neuroprotector homeobox (ADNP). [file 12917_2024_4148_MOESM4_ESM.zip › Suriyaphol - Suppl Fig. 1 Legend.docx]

**[Supplementary Fig. 1.](https://static-content.springer.com/esm/art%3A10.1186%2Fs12917-023-03824-9/MediaObjects/12917_2023_3824_MOESM1_ESM.docx)**

Partial least squares discriminant analysis (PLS-DA) plot depicting prominent proteins differentially expressed between non-metastatic (NmFMC) and metastatic feline mammary gland carcinoma (mFMC). **(A)** centromere protein F (CENPF). **(B)** erythrocyte membrane protein band 4.1 (EPB41). **(C)** trafficking kinesin protein 2 (TRAK2). **(D)** WD repeat domain 1(WDR1) **(E)** adenylate cyclase 10 (ADCY10). **(F)** activity-dependent neuroprotector homeobox (ADNP).
