# Supplementary figures and images for: Analysis of serum peptidome profiles of non-metastatic and metastatic feline mammary carcinoma using liquid chromatography-tandem mass spectrometry
Source: BMC Vet Res. 2024 Jun 29;20:280. doi: 10.1186/s12917-024-04148-y (PMC11218297; doi:10.1186/s12917-024-04148-y)

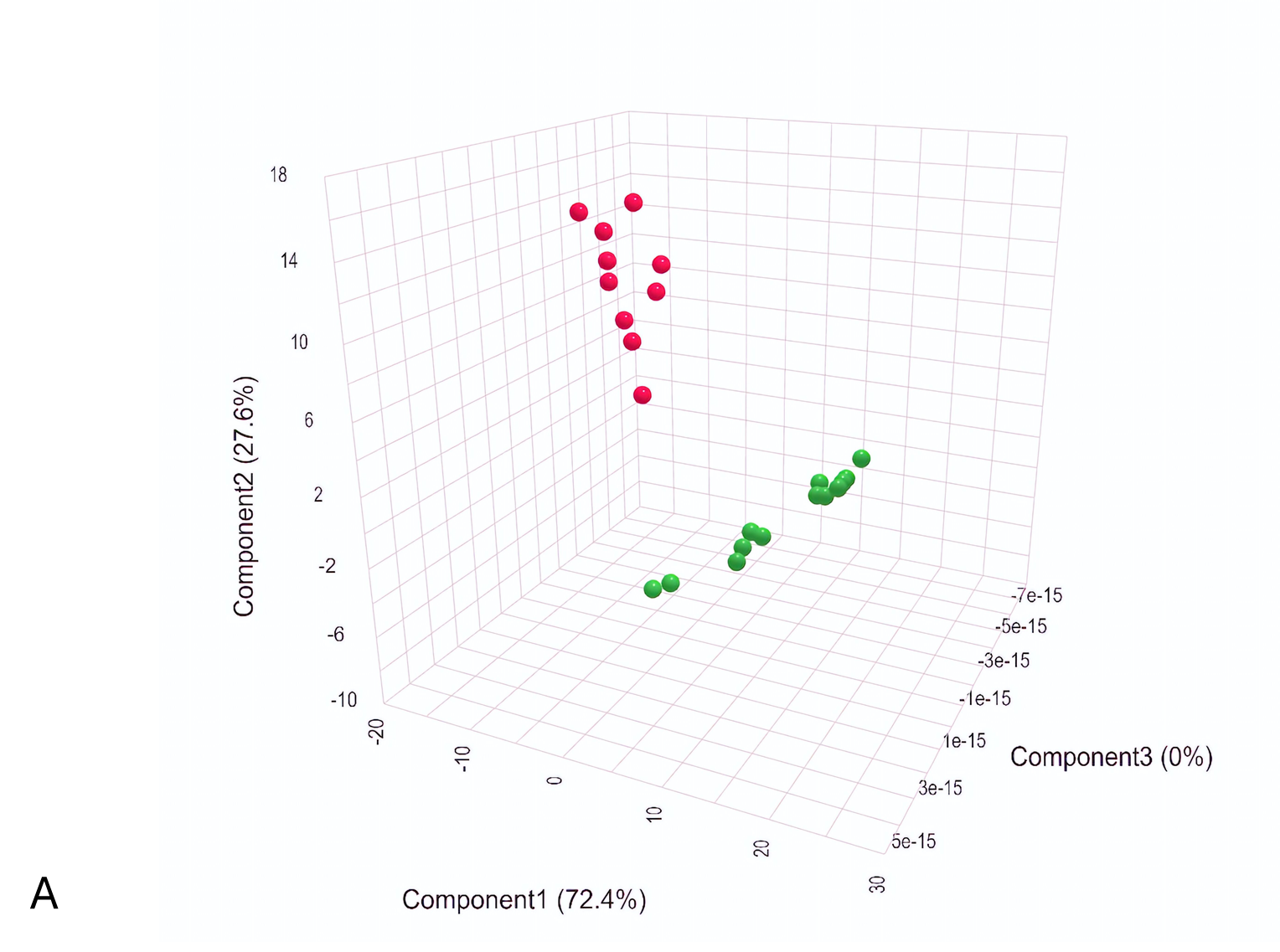

Supplement: Supplementary file 4 — Additional file 4: Supplementary Fig. 1. Partial least squares discriminant analysis (PLS-DA) plot depicting prominent proteins differentially expressed between non-metastatic (NmFMC) and metastatic feline mammary carcinoma (mFMC). (A) centromere protein F (CENPF). (B) erythrocyte membrane protein band 4.1 (EPB41). (C) trafficking kinesin protein 2 (TRAK2). (D) WD repeat domain 1(WDR1) (E) adenylate cyclase 10 (ADCY10). (F) activity-dependent neuroprotector homeobox (ADNP). [file 12917_2024_4148_MOESM4_ESM.zip › Suriyaphol - Suppl Fig. 1A - CENPF.tif]

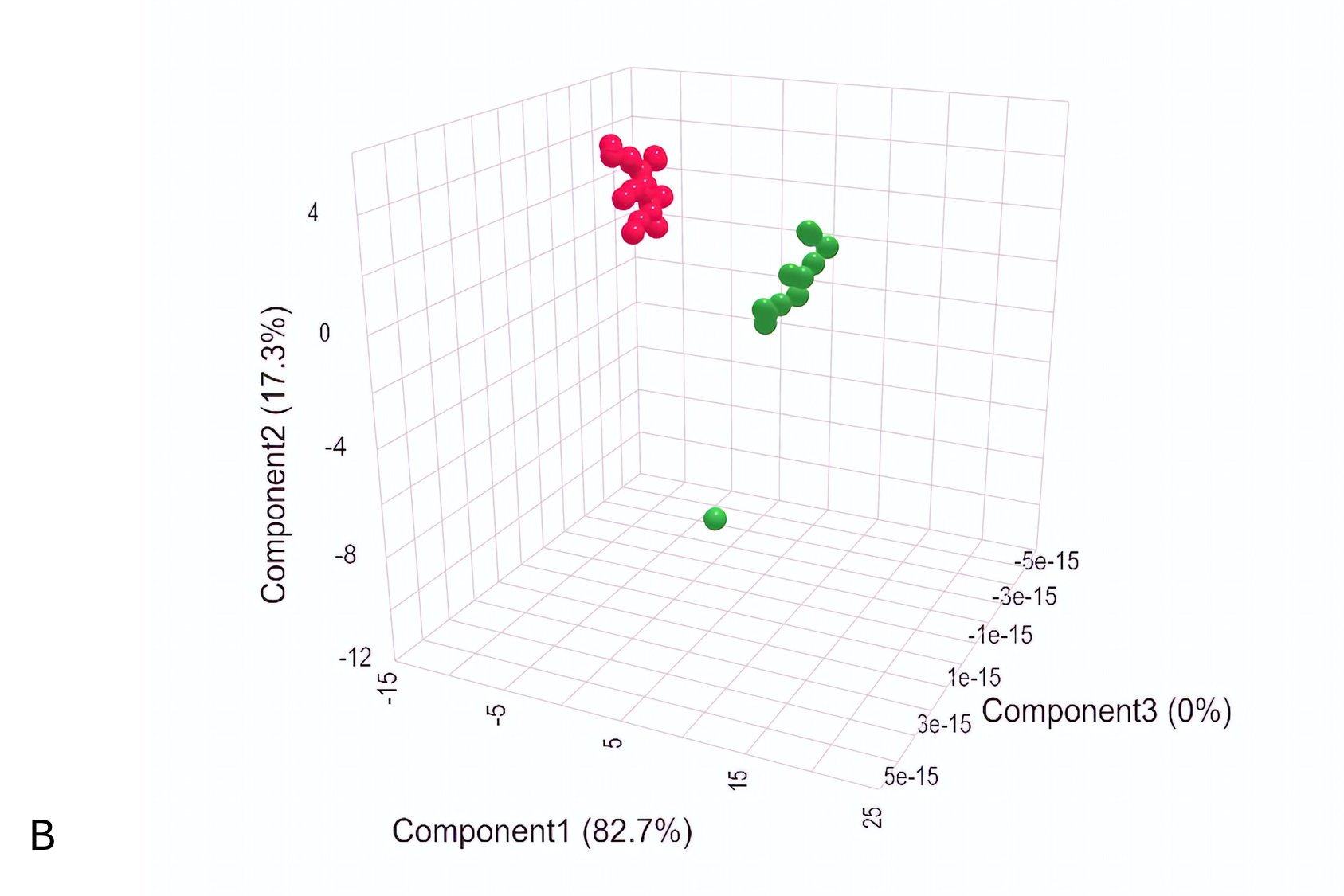

Supplement: Supplementary file 4 — Additional file 4: Supplementary Fig. 1. Partial least squares discriminant analysis (PLS-DA) plot depicting prominent proteins differentially expressed between non-metastatic (NmFMC) and metastatic feline mammary carcinoma (mFMC). (A) centromere protein F (CENPF). (B) erythrocyte membrane protein band 4.1 (EPB41). (C) trafficking kinesin protein 2 (TRAK2). (D) WD repeat domain 1(WDR1) (E) adenylate cyclase 10 (ADCY10). (F) activity-dependent neuroprotector homeobox (ADNP). [file 12917_2024_4148_MOESM4_ESM.zip › Suriyaphol - Suppl Fig. 1B - EPB41.tif]

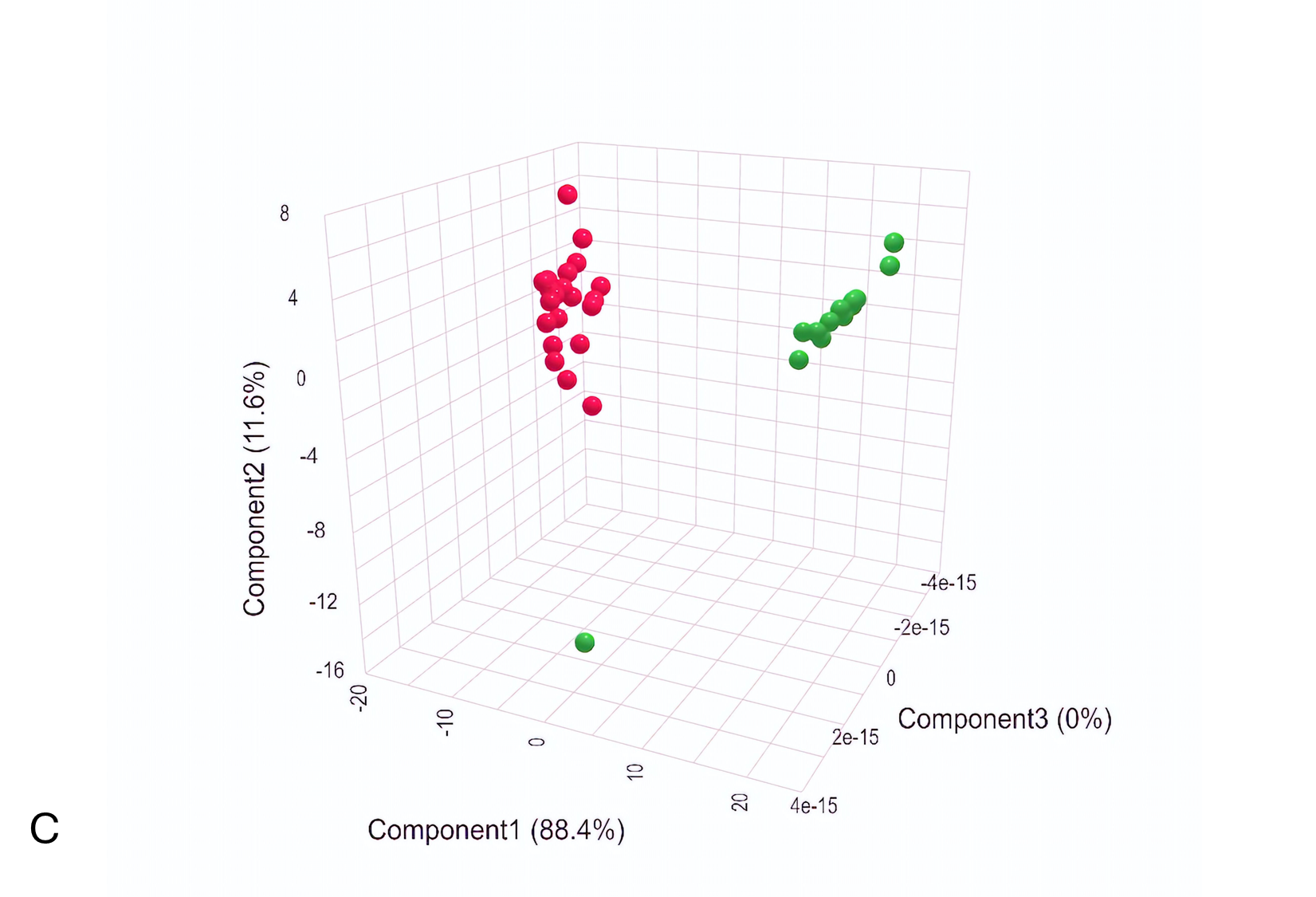

Supplement: Supplementary file 4 — Additional file 4: Supplementary Fig. 1. Partial least squares discriminant analysis (PLS-DA) plot depicting prominent proteins differentially expressed between non-metastatic (NmFMC) and metastatic feline mammary carcinoma (mFMC). (A) centromere protein F (CENPF). (B) erythrocyte membrane protein band 4.1 (EPB41). (C) trafficking kinesin protein 2 (TRAK2). (D) WD repeat domain 1(WDR1) (E) adenylate cyclase 10 (ADCY10). (F) activity-dependent neuroprotector homeobox (ADNP). [file 12917_2024_4148_MOESM4_ESM.zip › Suriyaphol - Suppl Fig. 1C - TRACK2.tif]

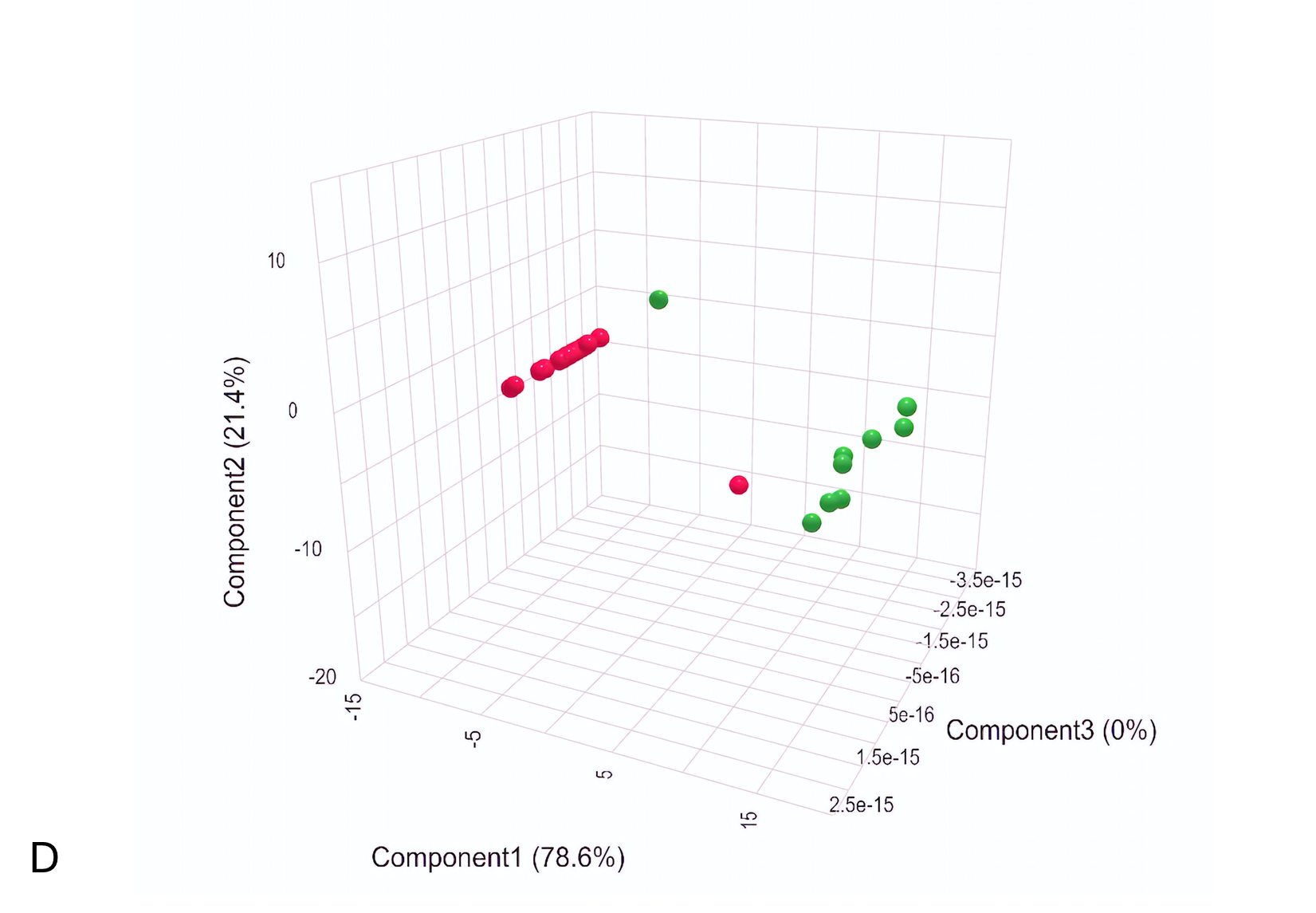

Supplement: Supplementary file 4 — Additional file 4: Supplementary Fig. 1. Partial least squares discriminant analysis (PLS-DA) plot depicting prominent proteins differentially expressed between non-metastatic (NmFMC) and metastatic feline mammary carcinoma (mFMC). (A) centromere protein F (CENPF). (B) erythrocyte membrane protein band 4.1 (EPB41). (C) trafficking kinesin protein 2 (TRAK2). (D) WD repeat domain 1(WDR1) (E) adenylate cyclase 10 (ADCY10). (F) activity-dependent neuroprotector homeobox (ADNP). [file 12917_2024_4148_MOESM4_ESM.zip › Suriyaphol - Suppl Fig. 1D - WDR1.tif]

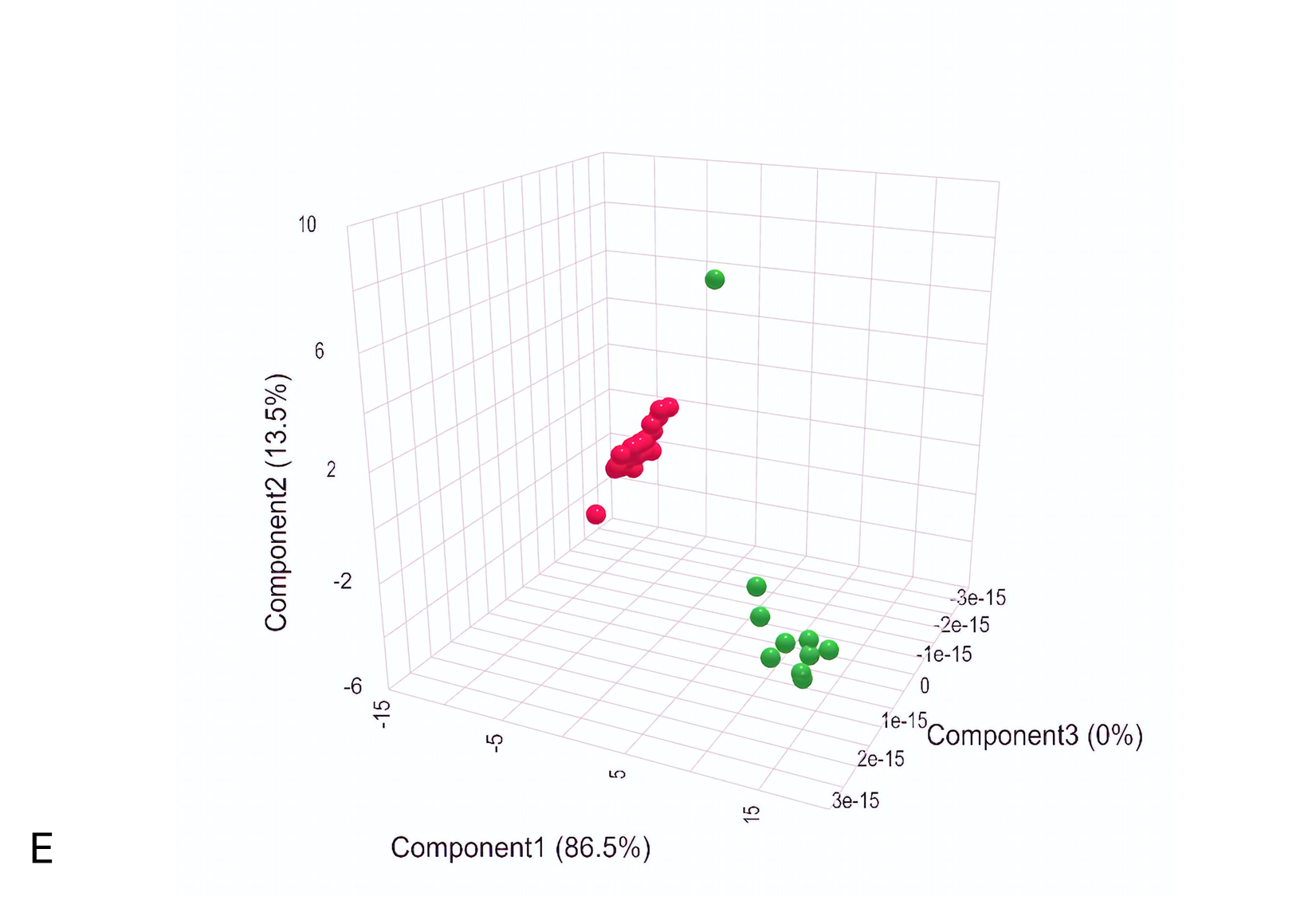

Supplement: Supplementary file 4 — Additional file 4: Supplementary Fig. 1. Partial least squares discriminant analysis (PLS-DA) plot depicting prominent proteins differentially expressed between non-metastatic (NmFMC) and metastatic feline mammary carcinoma (mFMC). (A) centromere protein F (CENPF). (B) erythrocyte membrane protein band 4.1 (EPB41). (C) trafficking kinesin protein 2 (TRAK2). (D) WD repeat domain 1(WDR1) (E) adenylate cyclase 10 (ADCY10). (F) activity-dependent neuroprotector homeobox (ADNP). [file 12917_2024_4148_MOESM4_ESM.zip › Suriyaphol - Suppl Fig. 1E - ADCY10.tif]

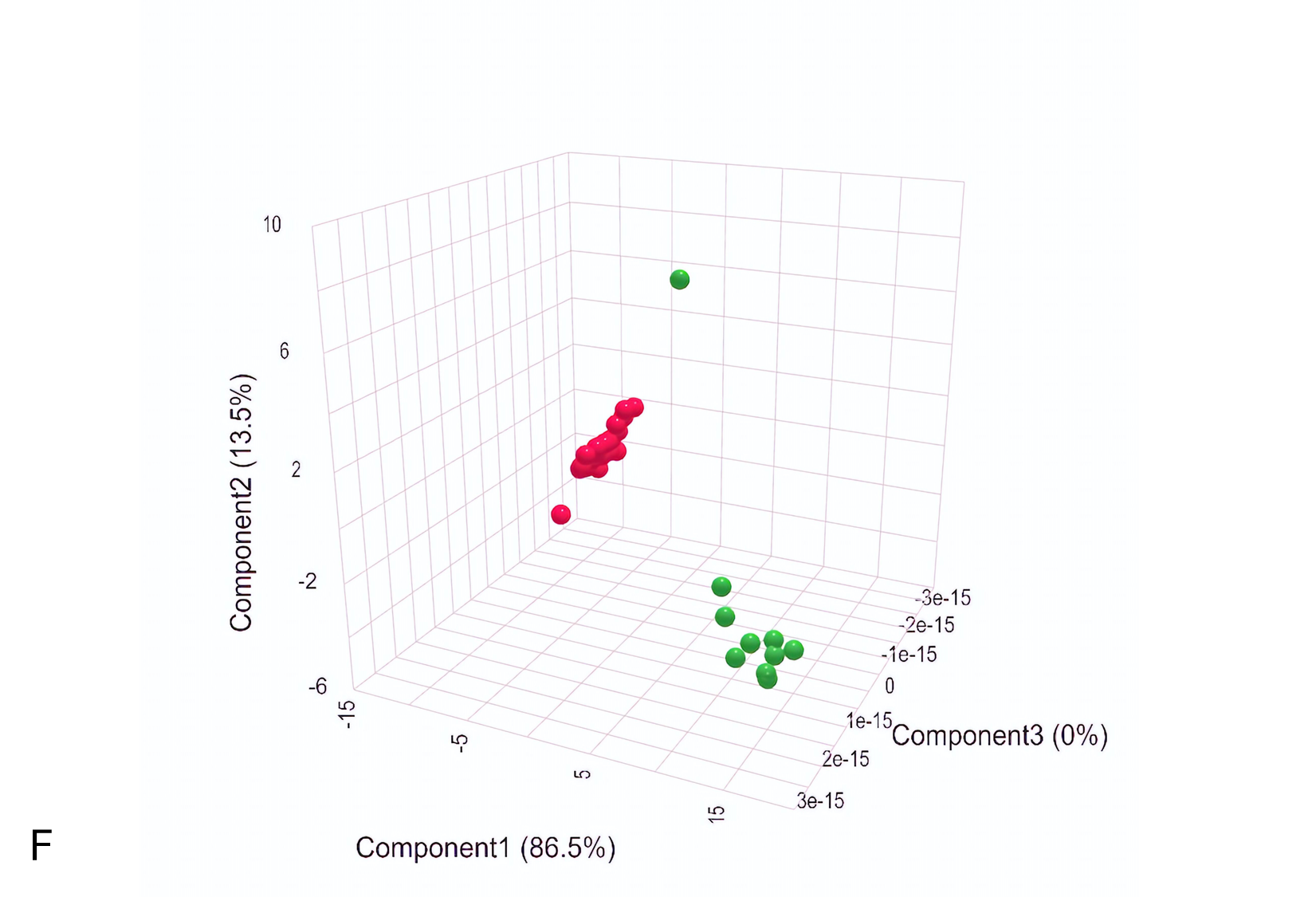

Supplement: Supplementary file 4 — Additional file 4: Supplementary Fig. 1. Partial least squares discriminant analysis (PLS-DA) plot depicting prominent proteins differentially expressed between non-metastatic (NmFMC) and metastatic feline mammary carcinoma (mFMC). (A) centromere protein F (CENPF). (B) erythrocyte membrane protein band 4.1 (EPB41). (C) trafficking kinesin protein 2 (TRAK2). (D) WD repeat domain 1(WDR1) (E) adenylate cyclase 10 (ADCY10). (F) activity-dependent neuroprotector homeobox (ADNP). [file 12917_2024_4148_MOESM4_ESM.zip › Suriyaphol - Suppl Fig. 1F - ADNP.tif]
